# Development of Reduced-Fat, Reduced-Sodium Semi-Hard Sheep Milk Cheese

**DOI:** 10.3390/foods8060204

**Published:** 2019-06-11

**Authors:** Golfo Moatsou, Evangelia Zoidou, Evangelia Choundala, Konstantinos Koutsaris, Olga Kopsia, Katerina Thergiaki, Lambros Sakkas

**Affiliations:** Laboratory of Dairy Research, Department of Food Science and Human Nutrition, Agricultural University of Athens, Iera Odos 75, 118 55 Athens, Greece; ezoidou@aua.gr (E.Z.); stud511028@aua.gr (E.C.); K_Koutsaris7@hotmail.com (K.K.); o.kopsia@gmail.com (O.K.); katerinather@hotmail.com (K.T.); lasakkas@hotmail.com (L.S.)

**Keywords:** sheep milk cheese, reduced-fat cheese, microparticulated whey protein (MWP), high-pasteurized cheese milk, sodium reduction, NaCl/KCl brine

## Abstract

This paper examines the effects of the incorporation of denatured whey proteins along with salting in NaCl/KCl brine on the characteristics and ripening of sheep milk reduced-fat (RF), semi-hard cheese. Incorporation of denatured whey proteins was carried out by: i. adding commercial microparticulated whey protein (MWP) in reduced-fat cheese milk (RFM), or ii. by ‘in situ’ heat-induced partial denaturation of whey proteins of reduced-fat cheese milk (RFD). The implemented cheesemaking conditions included curd washing, moderate clotting, scalding temperatures, and ripening of cheeses packed in plastic bags under vacuum at 10 °C. Full-fat cheeses (FF) were manufactured in parallel. Physicochemical composition, textural profile, and proteolysis were assessed throughout 60 days of ripening. The mean moisture, fat on dry matter (FDM), moisture on non-fat substances (MNFS), protein on dry matter (PDM), salt, and salt-in-moisture (S/M) content of the RF cheeses were 47.4%, 32.8%, 57.3%, 54.3%, 1.63%, and 3.36%, respectively; pH ≈ 5.0, a_w_ ≈ 0.977, Ca ≈ 1000 mg/100 g cheese. The MNFS of FF and RF cheeses were similar. Proteolysis indices were not affected by any of the treatments, and they were similar to the FF counterparts. The applied cheesemaking technology was adequate for the production of semi-hard reduced-fat and reduced-sodium cheeses. Ripening under packaging hindered moisture loss without impairing the evolution of proteolysis and textural parameters. The same holds true for salting in NaCl/KCl brine. The high pasteurization of cheese milk was more effective for the increase of moisture and MNFS than the addition of MWP, without exhibiting any adverse effects.

## 1. Introduction

Cheese is considered as a source of fat and sodium. The World Health Organization (WHO) [1,2] recommends: (i) the reduction of total and saturated fats intake to less than 30% and 10% of total energy intake, respectively, (ii) the reduction of sodium or salt intake to 2 g or 5 g per day, respectively, and (iii) the increase of potassium intake to at least 90 mmol or 3510 mg per day to achieve a molar ratio of potassium to sodium close to 1:1. Therefore, the development of reduced-fat and reduced-sodium cheeses is of great importance for both academia and industry.

The fat content of cheese milk apart from cheese yield affects dramatically the composition, the biochemistry of ripening, the microstructure, and the organoleptic/sensory properties of cheeses. The manufacture of reduced-fat or low-fat cheese requires interventions or modifications in cheesemaking technology in order to produce cheese with physical properties and moisture on non-fat substances (MNFS) acceptable by the consumers and comparable with the full-fat counterpart. The aim of interventions is to increase the moisture-to-protein ratio and decrease the aggregation of paracasein. They may be implemented in the cheesemilk—composition, heat treatment, homogenization, fat mimetics or replacers—and in the cheesemaking conditions—cutting, scalding and salting conditions, acidification, and specific adjunct starters [3,4,5,6]. 

The incorporation of denatured whey proteins into the cheese curd has been proposed to counteract fat reduction in cheese. In this respect, the fortification of reduced-fat/low-fat cheese milk with microparticulated whey protein (MWP) is practiced. MWP can act as a filler/fat mimetic within the high-protein/low-fat cheese mass without interacting with the other components [4,7,8,9]. Relevant research works have been published [10,11,12,13,14], whereas there are commercial products based on the denaturation of cow cheese whey proteins by heating. The incorporation of whey proteins into the curd can be achieved by the heat treatment of cheesemilk under conditions more severe than typical (low) pasteurization, which at first induces the denaturation of β-lactogloboulin. The result is the formation of the well-known micelle-bound β-lactogloboulin-κ-casein and soluble complexes of denatured whey proteins, which reduce syneresis and moisture loss from the curd [15,16]. However, extended heat denaturation of whey proteins in the cheese milk increases dramatically the rennet clotting time and results in weak, crumbly curds with high moisture [15]. In fact, denaturation up to 20% can be tolerated, since it results in satisfactory clotting time and syneresis level and in a 4% increase of cheese yield [17].

Salt is an essential cheese ingredient, which in addition to its direct contribution to flavor, affects the mineral composition of paracasein, decreases the moisture content and a_w_, influencing the growth of microorganisms and the activity of the enzymes in the cheese mass. As a result, it affects acidification and proteolysis, which in turn affect the cheese microstructure. Therefore, sodium reduction or substitution may have potentially adverse effects on cheese biochemistry, on the autolysis rate of starters, on cheese texture, and finally on the organoleptic properties [18,19,20]. Recent research studies on sodium reduction in various types of cheese have focused on the control of the physicochemical composition of the curd and the salting conditions [21,22,23,24,25,26], or the use of substitutes such as potassium chloride, salt-rich whey fractions, or flavor enhancers [23,27,28,29,30,31,32,33]. 

The objective of the present study was the development of a sheep milk reduced-fat, semi-hard cheese with improved characteristics. For this purpose, two research hypothesis were tested. The first was that the incorporation of denatured whey particles by means of a commercial MWP, or by the heat-induced partial denaturation of cheese milk whey proteins ‘in situ’, would improve the reduced-fat sheep milk characteristics. The second was that it would be possible to substantially reduce the cheese sodium content and partially replace it with potassium through salting.

## 2. Materials and Methods

### 2.1. Cheesemaking

Four different semi-hard cheeses were manufactured in triplicate from mixtures of full-fat (FF) and skimmed sheep milk, following the basic steps of the manufacture of Dutch-type varieties. FF cheese was made from sheep milk with 5.40 ± 0.046% fat and a protein-to-fat ratio (P/F) of 0.99 ± 0.030; it was pasteurized under batch conditions at 68 °C for 10 min. Reduced-fat (RF) cheese was made from milk with 2.98 ± 0.214% fat and P/F ratio 1.82 ± 0.168 pasteurized at 68 °C for 10 min. Reduced-fat cheese made from milk with 2.99 ± 0.252% fat fortified with 0.5% (*w*/*w*) microparticulated whey protein (MWP, Nutrilac CH-4560, Arla Foods, Viby, Denmark and a P/F ratio of 1.88 ± 0.196 was coded as RFM. Reduced-fat cheese from high-pasteurized milk heated at 72 °C for 10 min with 2.97 ± 0.238% fat and a P/F of 1.82 ± 0.167 was coded as RFD. A commercial starter that consisted of thermophilic and mesophilic strains (CHOOSIT AlpD, Danisco, DuPont Nutrition & Biosciences, Copenhagen, Denmark) was used according to the manufacturer’s instruction. Classical rennet (Naturen extra 1115NB, Chr. Hansen, Hoersholm, Denmark) was utilized at a ratio of 0.36 g per 10 kg of milk. After curdling at 31–32 °C, the curd was cut into 1–1.5 cm cubes. Curd washing and moderate scalding at 35–36 °C was performed. Molding and pressing were carried out by hand at room temperature. 

Fresh cheese remained in molds overnight. After the removal of molds, the small cheese wheels of 650–700 g were salted in brine bath at 10 °C for 4–4.5 h according to their weight. Half the cheese wheels were put in a 20% (*w*/*w*) classic NaCl brine (C) and the other half were put in a 20% (*w*/*w*) “light” NaCl/KCl 1:1 (L) brine. The cheese-to-brine ratio was 1:2 (*w*/*w*). Both brines were fortified with 0.3% (*w*/*w*) CaCl_2_ and pasteurized at 80 °C for 5 min. Therefore, eight cheeses resulted from each experimentation day. 

After brining, cheeses remained overnight at 17 °C to dry; then, they were transferred to a ripening room at 10 °C. Seven days after cheesemaking, they were packed in plastic bags under vacuum. Cheese ripening took place at 10 °C. Samples—each sample was a cheese wheel—were taken at seven, 30, and 60 days after cheesemaking, and coded by combining the code of the cheese milk and the code of the brine. 

### 2.2. Analyses

The gross composition of the full-fat cheese, skimmed milk cheese, and their mixtures was determined by a MilkoScan-FT1 Fourier Transform Infrared (FTIR) analyzer (Foss, Hilleroed, Denmark). The degree of β-lactoglobulin (β-lg) denaturation was estimated by means of reversed-phase high-performance liquid chromatography (RP-HPLC) [34]. 

At all the sampling points, the gross composition of cheeses was determined by means of a FoodScan-Dairy Near Infrared (NIR) analyzer (Foss, Hilleroed, Denmark). A dispersion of cheese in water was used for pH determination. The water activity (a_w_) was estimated using a bentchtop Dew Point Water Activity Meter (Aqualab). Texture profile analysis was carried out by means of the double bite test [35] using a Shimadzu Testing Instrument AGS-500 NG (Shimadzu Corporation, Kyoto, Japan). Proteolysis was assessed by estimating free amino groups using the trinitrobenzenesulphonic acid (TNBS) method [36] and by analyzing the water-soluble extract (WSE) of cheeses using RP-HPLC [37]. WSE was prepared as follows. Three g of cheese and 10 mL of ultra-pure water were homogenized by UltraTurrax (IKA-Werke GmbH & Co. KG, Staufen im Breisgau, Germany) at 9500 rpm for 2 min. After incubation at 40 °C for 60 min, homogenization was repeated. Then, cheese homogenate was centrifuged at 10,000× *g*, at 4 °C for 10 min. The top-fat layer was removed, and the supernatant was filtered through Whatman No. 42 filter paper. The filtrate was diluted with three parts of solvent A (0.1% trifluoroacetic acid (TFA) in ultra-pure water), and 100 µL of this dilution was injected into the column. 

Additional analyses of cheese mineral fraction were performed at day 60. The International Organization for Standardization / International Dairy Federation (ISO/IDF) potentiometric method [38] for the determination of chloride was utilized for the estimation of salt in cheese. Cheese ash preparation and the determination of calcium, magnesium, and potassium contents were performed according to the ISO/IDF reference method of the atomic absorption spectrometric method [39,40]. An organoleptic evaluation of 60-day-old cheese was carried out by a panel of five laboratory staff members who were familiar with cheese grading. Cheeses were presented in random order and they were graded for appearance, texture, and flavor on a 0 to 10-point scale. The three scores were multiplied by one, four, and five respectively, considering their relative contribution to the organoleptic acceptance of cheeses.

### 2.3. Statistical Analysis

Two-way analysis of variance (ANOVA) was applied to test the effects of: (i) treatments (the incorporation of denatured whey proteins and composition of brine), and (ii) the stage of ripening on the characteristics of reduced fat cheeses. Full-fat cheeses were not included in the analysis scheme because their much lower P/F ratio compared to reduced-fat cheeses could mask the effect of treatments. The differences among the means were tested using the least significance difference method (LSD) at *p* < 0.05. The software Statgraphics Centurion XVI (Manugistics, Inc., Rockville, MA, USA) was utilized.

## 3. Results

### 3.1. Cheese Milk, Cheesemaking Conditions, and Salting

The mean protein, fat content, pH, and β-lg reduction of cheese milks are presented in Table 1. The percentage reduction of native β-lg corresponds to its denaturation degree, and it was chosen as the most proper index for assessing the heating effect under low and high-pasteurization conditions [41]. Low pasteurization, which is typical for cheesemaking, cause a 2–4% denaturation of whey proteins, whereas up to 20% can be applied in cheesemaking as reported in the “Introduction” [17]. In our experiments, low-batch pasteurization at 68 °C for 10 min induced more than 4% denaturation of β-lg in sheep cheese milk. This effect could be due to the higher β-lg content of sheep milk compared to cow milk. Heating at 72 °C for 10 min caused a reduction of native β-lg by 28%, which is approximately 20% more than low pasteurization. It has to be noticed that this temperature/time combination has been selected from preliminary experiments aiming to achieve 25–30% denaturation of sheep milk β-lg under batch heating conditions. Coagulation time was not significantly affected (*p* > 0.05) by any of the experimental factors; however, a higher but inconsistent mean clotting time was observed for high-pasteurized RF cheese milk due to the effect of the β-lg/κ-casein complex bound onto the casein micelle [15]. 

The cheesemaking conditions and weight loss of cheeses after salting and overnight drying are summarized in Table 2. The variability of stirring in terms of duration resulted from the variability of curdling and clotting time. Curd washing, i.e. the partial substitution of whey with water, and the moderate clotting/curdling and scalding temperatures are characteristic features of the manufacture of Dutch-type varieties. Therefore, the findings of the present study are often discussed in relation to this cheese group.

According to Table 2, both treatments of RF cheese milk increased statistically significantly (*p* < 0.05) the yield, expressed as fresh unsalted cheese. The most profound increase resulted from the controlled denaturation of β-lg (RFD). 

Salt intake takes place at the aqueous phase of cheese, and results in the loss of approximately double the quantity of water, since the size of Na^+^ and Cl^−^ ions is approximately twice that of H^+^ and OH^−^. Salt diffusion in the cheese mass is affected mainly by three factors [18,19]: (i) the pore size of the paracasein matrix, (ii) the presence of soluble/charged substances—such as organic acids, nitrogenous compounds, and minerals—which increase the density of the cheese water-soluble fraction and hinder the movement of NaCl, and (iii) milk fat globules and hydrated casein particles forcing salt molecules to non-linear routes. The weight loss during salting (Table 2) is consistent with the above-mentioned phenomena. In particular, the losses in full-fat (FF) cheeses with much higher numbers of milk fat globules were lower—although not significantly—for both C and L brines. The same holds true for LFD cheeses from high-pasteurized milk. Apparently, the size of paracasein micelles increased due to the connection with denatured WP. Also, most of the L-cheeses exhibited lower weight loss. This behavior could be attributed to a higher atomic radius of K—227 pm—than that of Na—186 pm—which could retard its movement. Moreover, the higher molecular mass of K, ~39 g/mol versus 23 g/mol, implies that brine L contained fewer salt molecules than C.

A rapid decrease of brine pH was observed within the first hour of salting, which was apparently due to the migration of lactic acid from the cheese mass to the brine. The mean initial pH of brines C and L was 7.19 ± 0.198 and 7.68 ± 0.035, respectively. The respective mean pH after one hour of cheese salting decreased to 5.54 ± 0.088 and 5.73 ± 0.053, whereas it remained almost constant thereafter; after four hours, the respective pH values were 5.41 ± 0.075 and 5.58 ± 0.039. 

### 3.2. Characteristics of Cheeses

The composition and textural profile of the experimental sheep milk cheeses are shown in Table 3. Full fat FF-C and FF-L cheeses were not included in the scheme of the statistical analysis to avoid the interference of their much higher fat content. Therefore, RF cheeses were the actual control of the experiments. 

The comparison of mean FDM contents of FF and RF cheeses (Table 3) indicate that RF cheeses are consistent with the European Union (EU) regulation on nutrition and health claims made on foods [42], which lays down the claim that “reduced-fat” may only be made where the reduction in content is at least 30% compared to a similar product.

Most of the compositional and textural parameters were significantly (*p* < 0.05) affected by the ripening. MNFS and a_w_ decreased in parallel to moisture loss, which decreased by approximately 1% during two months of ripening as a result of packaging. pH was significantly increased from day seven to day 30. All the RF cheeses that had an approximate pH value of 5.0 were affected significantly (*p* < 0.5) by the treatments of cheese milk or the brine composition. The increase of pH in the first weeks of ripening (Table 3)—it did not change thereafter—cannot be attributed to proteolysis, but rather to changes in the salt equilibria within the RF cheese mass due to its high protein and mineral content. The inclusion of denatured whey proteins by the heat treatment of cheese milk (RFD cheeses) resulted in the highest moisture due to reduced syneresis [15,16,17].

The water activity (a_w_) of our cheeses was not affected by NaCl substitution andthe treatments of RF cheese milk (Table 3). As mentioned previously, the main steps of the cheesemakings of the present study are applied during the manufacture of Dutch-type varieties [43]. The water activity (a_w_) of this cheese category ranges from 0.95 to 0.97, whereas the typical pH of Edam is approximately pH 5.7, and the typical pH of Gouda cheese is 5.2–5.3; in fact, the pH of this group can be anywhere within the range of pH 5.0 and 5.6 [19,20,43,44]. The MNFS of the cheeses of the present study was within the range of 53% to 63% reported for Dutch-type varieties [43]. The differences indicated by the statistical analysis were marginal; in fact, they are due to the 0.47% difference between RF and RFD cheeses, which is very close to the respective LSD value (0.42%). The finding that the a_w_ and MNFS of FF and RF cheeses were similar is in accordance with a main objective of the manufacture of reduced-fat cheeses [5].

It was evident that all the reduced-fat cheeses (RF) were much harder than their full-fat counterparts (FF), which was apparently due to the higher PDM and Ca contents and despite their higher moisture [45]. In brief, the parameters of textural profile analysis are the following [35,46]. Hardness or firmness (N) is the force necessary to attain deformation of the food matrix during the first bite. Cohesiveness is the ratio of positive area of the second compression to that observed during the first compression, and expresses the degree to which the chewed mass holds together. Adhesiveness (N × mm) is the work that is needed to overcome the attractive forces in the protein matrix, and expresses the degree to which the chewed mass sticks to mouth surfaces. Gumminess (N) is the product of firmness and cohesiveness, and expresses the energy required to disintegrate the food to a state ready for swallowing. The hardness of the present cheeses was not significantly affected by ripening, opposite to the cohesiveness, adhesiveness, and gumminess. 

The salt, calcium, magnesium, and potassium contents of mature 60-day-old cheeses are presented in Table 4. The treatment of RF cheese milk and the composition of brine did not affect salt intake as indicated by the salt-in-moisture (S/M) content. The Ca and Mg contents of RF cheeses were high—close to 1000 mg and 50 mg per 100-g cheese, respectively—and higher than FFin accordance to their higher protein/fat ratio, as both Ca and Mg are parts of the casein micelle complex. Moreover, the salt and S/M contents of FF and RF cheeses were similar, indicating that the increase of the number or the size of protein particles in RF cheeses counteracted the effect of higher counts of milk fat globules in FF cheeses. As expected, the composition of brine dramatically affected the K content of cheeses; those salted in brine L contained more than 2.7 times more potassium than their C counterparts. The salt and S/M content of the cheeses of the present study were <1.7% and <3.5%, respectively, which are lower than those in similar cheeses. Salt 2–2.4% and S/M content 4.7–4.9% are reported for typical Edam and Gouda with 41–43% moisture [19,47]. The EU regulation on nutrition and health claims made on foods [42] lays down the claim that “reduced-sodium” may only be made where the reduction in content is at least 25% compared to a similar product. Accordingly, cheeses salted in NaCl/KCl brine (L), are reduced-sodium cheeses. Moreover, the low content of cheeses salted in classical brine (C) can be also characterized as reduced-sodium compared to similar Dutch-type cheeses.

The proteolysis indices are shown in Table 5. The concentration of free amino groups expressed as mM Gly increased statistically significantly (*p* < 0.05) from day 7 to day 60 due to the hydrolysis of residual caseins and peptides in the cheese mass. The RP-HPLC profiles of the WSEs of the cheeses were divided into four parts according to the elution time, and the area of each part was calculated as previously reported [48]. The area of the 0–10 min part is not presented, because it consisted of compounds that were not retained onto the column, such as free amino acids and other non-protein nitrogenous and inorganic substances. The rear 70–100 min part included hydrophobic and/or large peptides and native whey proteins. The concentration of free amino groups and the partial areas were not affected significantly (*p* > 0.05) by the treatment of RF cheese milk and by the brine composition, and they were similar to their full-fat counterparts. On the other hand, the ripening time significantly increased (*p* < 0.05) the free amino acids and hydrophilic small and medium sized peptides eluted within 10–70 min. The index HB/HL, i.e. the hydrophobic to hydrophilic peptides, expresses the ratio of the areas of part 55–100 to 10–55 min, and it has been proposed as an index of the course of proteolysis in various cheese categories [48]. From Table 5, it is evident that ripening markedly decreased this ratio, indicating the hydrolysis of hydrophobic/large peptides of the rear part of the RP-HPLC profiles. Therefore, under the particular ripening conditions significant proteolysis changes were observed, as happened with cohesiveness, adhesiveness and gumminess (Table 3). Under the conditions of cheesemaking (e.g. low scalding) and the physicochemical environment of the present cheeses (low pH, high moisture), residual rennet was likely the main proteolytic factor, which is consistent with the significant increase of the 10–55 min part of the RP-HPLC profiles during ripening [48].

The results of the statistical analysis of organoleptic evaluation of cheeses is shown in Table 6. In this case, FF cheese was included in the scheme. The control RF cheese obtained the significantly (*p* < 0.05) highest score in flavor, and all the reduced-fat cheeses were significantly better than their FF counterparts in terms of appearance and texture. The control RF was the most appreciated, followed by RFD and RFM. Finally, the salting in NaCl/KCl brine decreased the flavor and texture scores. Metallic taste or bitter or any other off-flavor were not reported for any of the present cheeses, which was in accordance with the proteolysis course (Table 5).

## 4. Discussion

The experimental factors of the present study induced sporadic statistically significant differences between reduced-fat, semi-hard sheep milk RF cheeses. The low salt content of the present cheeses resulted in higher a_w_ and lower pH than those reported for their Dutch-type counterparts (Table 3 and Table 4). Salt intake is the main factor responsible for the reduction of a_w_. It expresses the water that is available for the reactions and microbial growth, being as important as pH and temperature [20]. In fresh cheeses, a_w_ is configured almost exclusively by the salt content. In the progress of cheese ripening, new molecules such as lactic acid, peptides, and calcium phosphate are produced that bind water molecules and further reduce the a_w_ [19]. Therefore, the low salt-in-moisture (S/M) content and the high a_w_ at the first days of ripening of the present cheeses did not impair the growth of the starter and the accumulation of lactic acid.

The textural properties of cheese and in particular hardness are configured in two stages. The first stage is the weakening of curd due to the solubilization of colloidal calcium phosphate induced by acid development during the first days or even weeks of ripening [45]. For example, during the first three to four weeks of Cheddar ripening, approximately 15–18% of insoluble Ca is solubilized [49]. The 71% of calcium has been found in the soluble fraction in soft-type Feta cheese, which undergoes intense acidification during the first two to three days after manufactureopposite to the 25% observed in hard-type Gruyere-type Graviera cheese [50]. The second stage is controlled mainly by proteolysis provided that no significant pH change occurs. Gradual changes of textural properties are observed in this stage, resulting from the binding of free water by the NH_3_^+^ and COO^-^ groups of the new peptides [49], which was confirmed by changes in cohesiveness, adhesiveness and gumminess of the present cheeses. Although RF milk treatments did not affect hardness, RFM cheeses with the addition of MWP had significantly lower cohesiveness and gumminess compared to the control RF cheeses. MWP can weaken the low-fat cheese structure, causing a casein dilution effect that results in better flow and melting behavior [7,10]. 

The composition of the brine did not affect significantly moisture and a_w_, and consequently the pH, which are main criteria for the selection of salting conditions of cheese. Apart from composition, the textural parameters were not affected either, which is in accordance with the majority of publications on sodium reduction or substitution in various cheese varieties; some of these findings are presented below. The reduction of salt content of Cheddar cheese from 1.8% to 1.3% under constant moisture levels did not affect bacterial counts, proteolysis, and the activity of ripening enzymes [21]. Moreover, the salt content reduction of the same cheese from 1.80% to 1.25% affected statistically significantly only the moisture on non-fat substances (MNFS), but not proteolysis, lipolysis, and acidification [22]. KCl has been used successfully to reduce the sodium content of Cheddar from 660 mg/100 g to 350 mg/100 g without changing moisture content, a_w_, and texture profile [51]. Similarly, the reduction of S/M content by 25%, i.e. from 4.25% to 3.09% in semi-hard Prato cheese did not affect significantly the gross composition, firmness, peptides profile and, sensory acceptance [25]. The reduction of NaCl by 30% from 1.28/100 g to 0.919/100 g by means of reducing the salting duration of semi-hard Tyba cheese, increased sporadically and marginally the moisture, pH, and ripening index throughout 40 days of ripening, but it significantly affected the textural parameters [26]. The salting of semi-hard Prato cheese using 60% NaCl + 40% KCl and sodium reduction from 667 mg/100g to 479 mg/100g had no effect on the moisture, MNFS, a_w_, pH, texture profile, flavor, and melting behavior [32]. On the other hand, the use of NaCl/KCl 1:1 mixture for salting of Prato cheese [33] resulted in significantly higher pH and proteolysis during ripening compared to control cheeses without influencing the diffusion of water molecules into the cheese matrix. The use of NaCl/KCl brine at a ratio of 2:1, on a molecular basis, did not influence pH and proteolysis expressed as mM glycine or as the sum of 15 free amino acids, and did not markedly change the sensorial, textural, biochemical, or microbiological features of Gouda cheese [23]. Sodium substitution has been also studied in brined cheeses. Salting of Feta and with mixtures NaCl/KCl 3:1 and 1:1 did not affect the composition, proteolysis, lipolysis, and textural properties during ripening [27,28]. Soluble nitrogenous fractions were not influenced by the storage of Halloumi cheese for eight weeks in various NaCl/KCl brines with ratios varying from 3:1 to 1:3 [29]. The substitution of NaCl by KCl up to a 1:3 ratio did not alter the gross composition, texture profile, and organoleptic features of white brined Akawi cheese, whereas substitution up to 1:1 did not affect significantly various proteolysis indices during cheese ripening; on the contrary, pH significantly increased when NaCl substitution took place [30]. 

To our knowledge, either the addition of MWP or partial WP denaturation have been scarcely reported for sheep milk cheeses. There are several publications for the use of microparticulated whey protein (MWP) in reduced-fat or low-fat semi-hard cheeses from cow milk. The addition of MWP at rather high levels of 3% or 6% in cow cheese milk with 1% fat, caused—after six weeks of ripening of Norvegia Gouda-type cheese—an increase of moisture from 49.2% to 51.26% and 52.28% respectively; whereas the 6% addition slightly increased S/M. Moreover, according to texture profile and sensory analyses, the addition of MWP resulted in soft cheese, which was not well accepted when 6% MWP was added [52]. The addition of 1% MWP in the cheese milk of a semi-hard cheese with an FDM similar to the present study adjusted the hardness and gumminess to levels similar to the full-fat counterpart, by weakening the paracasein network. The moisture content increased statistically significantly from 51% to 52.4%, while the MNFS was similar to the full-fat control, and proteolysis was not affected. More pronounced effects are reported for similar low-fat cheese with 5.6% FDM [10]. The use of various MWPs at 1% in pasta-filata Kashar cheese with 16.4% FDM caused a substantial increase of moisture and MNFS from 55.24% to 59.20% and from 59.60% to 63.43%, respectively; the latter was similar to its full-fat counterpart. All the textural parameters were statistically significantly changed, and one particular MWP caused more than a 50% reduction of hardness along with a substantial improvement of the overall organoleptic quality [53]. Recently, the addition of microparticles of denatured whey protein in full-fat sheep milk, which induced a 10% moisture increase, has been presented [14]. The use of MWP in our experiments with sheep milk at the recommended level of 0.5% did not cause composition or proteolysis changes, but did affect the texture profile by reducing the cohesiveness and gumminess. Therefore, our results confirm those of Drake and Swanson [54], who concluded that in cheeses with a fat reduction of 25–33%, the use of MWP does not seem necessary, because an increase of cheese moisture and yield can be achieved with modifications of cheesemaking conditions.

Half-fat Cheddar cheeses were manufactured from milk treated at 72 °C, 77 °C, 82 °C, and 87 °C for 26 s. The combination 82 °C/26 s caused 20% denaturation of the whey proteins and statistically significantly increased the cheese moisture and MNFS compared to the control treatment at 72 °C, which is in accordance to our findings. Moreover, similarly to the present study, the cheese pH was lower throughout ripening, which was attributed to higher moisture/MNFS contents and the concomitant lower buffering capacity. Higher MNFS also favored the formation of higher quantities of water-soluble nitrogen in high-pasteurized cheeses, while the small peptides and amino acids content was lower compared to its low-pasteurized counterpart. The cheese hardness reduced as the heating temperature increased, and the conclusion was that increasing the temperature from 72 °C to 77 °C, corresponding to a 2.8% and 8.4% denaturation of whey proteins, has minimal effects on cheese [55]. The heating of full-fat Gouda cheese milk at 80 °C for 60 s under pilot scale conditions induced 22 ± 1.8% β-lg denaturation in the cheese milk, which is close to our results. Heat treatment at 80 °C and 85 °C for 60 s increased the protein transfer from milk to curd by 6% and the β-lg content of Gouda cheese from <1% to 1.7% and 4.5%, respectively. A delay in rennet coagulation and the retardation of proteolysis compared to the pasteurized control was observed [56]. To our knowledge, high pasteurization as an intervention for reduced-fat sheep cheese milk has been applied only in Halloumi cheese [57]. According to the results, the treatment of cheese milk at 75 °C for 10 min significantly increased the cheese yield, moisture, and PDM by 20%, 12%, and 2.5%, respectively. Moreover, all the parameters of texture profile analysis were substantially significantly reduced, and the scores of organoleptic evaluations were significantly higher.

In the present study, reduced-fat cheeses as a group exhibited statistically significantly higher organoleptic scores compared to those of the full-fat counterparts, whereas sodium substitution by potassium resulted in lower scores. It is considered that the metallic or bitter taste of KCl limits its use to moderate levels of substitution. Alternative sources of potassium with the simultaneous use of flavor enhancers have been utilized to substitute 75% of sodium chloride in Cheddar cheese [31]. Nevertheless, considering that i. bitterness or metallic taste were not detected, ii. cheese characteristics were marginally or not impaired, and iii. the sodium-to-potassium ratio was favorable, it is concluded that salting in NaCl/KCl brine along with fat reduction can be applied for the manufacture of semi-hard sheep milk cheeses with improved nutritional characteristics.

## 5. Conclusions

The cheesemaking technology applied in the present study was adequate for the production of semi-hard reduced-fat/reduced-sodium cheeses with a_w_ and MNFS values similar to their full-fat counterparts. Ripening under packaging hindered moisture loss and did not impair the evolution of proteolysis and textural changes. The same holds true for the salting in NaCl/KCl brine. According to the results, the high pasteurization of cheese milk was more effective for the increase of moisture and MNFS compared to the addition of MWP, without exhibiting any adverse effects. 

## Figures and Tables

**Table 1:** Characteristics of sheep cheese milk. Mean of three experiments ± standard deviation. β-lg: β-lactoglobulin; FF: pasteurized (68 °C for 10 min) full-fat milk; RF: pasteurized reduced-fat milk; RFM: pasteurized reduced-fat milk supplemented with microparticulated whey protein (MWP); RFD: reduced-fat milk heated at 72 °C for 10 min. ^1^

Sheep Milk	FF	RF	RFM	RFD
Fat, %	5.40 ± 0.046	2.98 ± 0.214	2.99 ± 0.252	2.97 ± 0.238
Protein, %	5.33 ± 0.096	5.40 ± 0.103	5.61 ± 0.098	5.39 ± 0.078
Protein/fat	0.99 ± 0.030	1.82 ± 0.168	1.88 ± 0.196	1.82 ± 0.167
pH	6.59 ± 0.096	6.60 ± 0.073	6.63 ± 0.054	6.63 ± 0.054
% reduction of native β-lg	8.4 ± 2.69	9.6 ± 2.47 ^a^	5.9 ± 3.39 ^a^	28.4 ± 4.38 ^b^

^1^ FF milk was not included in the statistical analysis. ^a, b^ Different letters within rows indicate statistically significant differences.

**Table 2:** Manufacturing conditions of sheep milk semi-hard cheeses considering the addition of rennet as the starting point. The starter was added approximately 10 min before the addition of rennet. Means of three experiments ± standard deviation. Codes are according to Table 1. C: 20% (*w*/*w*) NaCl brine; L: 20% (*w*/*w*) NaCl/KCl 1:1 brine. ^1^

Stage	FF	RF	RFM	RFD
Coagulation, min after renneting	11 ± 0	11.7 ± 0.58	11.7 ± 0.58	12.7 ± 1.16
Cutting, min after renneting	44.7 ± 6.35	43.3 ± 4.04	43.0 ± 5.20	44.3 ± 5.13
Resting period, min after cutting	3 ± 0	3 ± 1	4 ± 1.7	3.7 ± 1.5
Duration of first stirring, min	19 ± 7	18 ± 3	16 ± 5	16 ± 1
Partial Replacement of Whey with Water				
Duration of second stirring, min	18 ± 6	17 ± 4	17 ± 7	14 ± 2
Resting for 5 min				
Molding				
Yield on the next day, % ^2^	20 ± 0.3	17.2 ± 0.3 ^a^	17.8 ± 0.3 ^b^	18.2 ± 0.2 ^c^
Salting				
% weight loss in brine C ^3^	5.1 ± 0.44	5.4 ± 0.56	5.3 ± 0.65	5.1 ± 0.06
% weight loss in brine L ^3^	4.8 ± 0.13	5.4 ± 0.52	5.1 ± 0.14	4.7 ± 0.77

^1^ FF cheeses were not included in the statistical analysis. ^2^ Yield was expressed as percentage of non-salted cheese weight on cheese milk weight. ^3^ Cheeses were weighed after salting for 4.0–4.5 h and overnight drying at 17 °C. ^a–c^ Different letters within rows indicate statistically significant differences.

**Table 3:** Compositional and textural characteristics of reduced-fat sheep milk cheeses. Cheese codes are according to Table 1 and Table 2. SE: standard error; LSD: least significant difference; MOI: moisture; FDM: fat on dry matter; MNFS: moisture on non-fat substances; PDM: protein on dry matter; C: classic NaCl brine; L: “light” NaCl/KCl 1:1 brine. ^1^

Cheese	pH	a_w_	MOI, %	FDM, %	MNFS, %	PDM, %	Hardness, N	Cohesiveness	Adhesiveness, N × mm	Gumminess, N
FF-C (*n* = 9)	4.92	0.976	42.78	48.07	59.00	42.82	9.33	0.422	82.96	3.93
FF-L (*n* = 9)	4.88	0.975	43.45	47.83	59.56	42.88	8.91	0.441	83.14	3.68
Reduced-Fat Cheeses										
Mean (n = 54)	4.97	0.977	47.38	32.83	57.27	54.26	11.45	0.431	109.39	4.93
RF-C (*n* = 9)	5.03 ^b^	0.979	46.80 ^a^	33.63	57.03 ^a^	54.80	11.46	0.467 ^b^	113.75	5.41 ^b^
RF-L (*n* = 9)	5.01 ^a,b^	0.977	47.23 ^a,b^	33.40	57.34 ^a,b^	54.52	12.15	0.420 ^a,b^	117.13	5.16 ^b^
RFM-C (*n* = 9)	4.95 ^a^	0.977	47.27 ^a,b^	32.90	57.20 ^a,b^	53.69	11.35	0.399 ^a^	112.15	4.63 ^a,b^
RFM-L (*n* = 9)	5.00 ^a,b^	0.976	47.44 ^a,b^	32.76	57.31 ^a,b^	53.93	10.52	0.402 ^a^	99.63	4.16 ^a^
RFD-C (*n* = 9)	4.97 ^a^	0.978	47.90 ^b^	32.03	57.50 ^b^	54.28	11.67	0.444 ^a,b^	102.31	4.98 ^a,b^
RFD-L (*n* = 9)	4.97 ^a^	0.977	47.67 ^b^	32.24	57.35 ^a,b^	54.35	11.56	0.452 ^a,b^	111.37	5.22 ^b^
SE	0.020	0.0012	0.234	0.581	0.146	0.598	0.655	0.0196	7.114	0.310
LSD	0.058	0.0035	0.693	1.667	0.420	1.715	1.864	0.0559	20.251	0.8820
Days of Ripening										
7 (*n* = 18)	4.94 ^a^	0.983 ^c^	47.91 ^c^	33.63 ^b^	58.09 ^a^	53.17 ^a^	11.42	0.543 ^c^	57.71 ^a^	6.22 ^c^
30 (*n* = 18)	5.01 ^b^	0.977 ^b^	47.39 ^b^	32.53 ^a,b^	57.20 ^b^	55.25 ^b^	11.69	0.404 ^b^	130.47 ^b^	4.77 ^b^
60 (*n* = 18)	5.02 ^b^	0.973 ^a^	46.85 ^a^	32.32 ^a^	56.57 ^c^	54.37 ^a,b^	11.26	0.345 ^a^	140.18 ^b^	3.79 ^a^
SE	0.014	0.0009	0.165	0.411	0.103	1.036	0.463	0.0139	5.030	0.226
LSD	0.033	0.0025	0.483	1.179	0.295	1.212	1.318	0.0401	14.320	0.633

^1^ FF cheeses were not included in the statistical analysis. ^a–c^ Different letters within columns indicate statistically significant differences.

**Table 4:** Salt (NaCl or NACl/KCl) and particular mineral content of reduced-fat sheep milk cheeses at 60 days of ripening. Cheese codes are according to Table 1 and Table 2. S/M: salt-in-moisture = (%salt × 100)/(%salt + %moisture); SE: standard error; LSD: least significant difference. ^1^

Cheese	Salt, %	S/M, %	Ca, mg/100 g	Mg, mg/100 g	K, mg/100 g
FF-C (*n* = 3)	1.50	3.39	862	43.15	78.90
FF-L (*n* = 3)	1.52	3.45	887	44.70	265.55
Reduced-fat cheeses					
Mean (*n* = 18)	1.63	3.36	981	49.79	
RF-C (*n* = 3)	1.53	3.23	983	50.75	85.70 ^a^
RF-L (*n* = 3)	1.55	3.20	1008	50.13	245.25 ^b^
RFM-C (*n* = 3)	1.61	3.34	981	50.0	97.75 ^a^
RFM-L (*n* = 3)	1.64	3.39	993	48.97	260 ^b,c^
RFD-C (*n* = 3)	1.69	3.44	969	50.35	96.25 ^a^
RFD-L (*n* = 3)	1.74	3.54	956	49.10	274.40 ^c^
SE	0.070	0.138	41.7	2.318	7.165
LSD	0.217	0.426	128.4	6.770	24.795

^1^ FF cheeses were not included in the statistical analysis. ^a–c^ Different letters within columns indicate statistically significant differences.

**Table 5:** Proteolysis indices of reduced-fat sheep milk cheeses. Cheese codes are according to Table 1 and Table 2. HB/HL: hydrophobic to hydrophilic peptides, i.e. the ratio of the areas of parts 55–100 to 10–55 min. ^1^

Cheese	mM Gly	Parts of RP-HPLC Profiles (% Area on Total Profile Area)			
		10–40 min	40–70 min	70–100 min	HB/HL
FF-C (*n* = 9)	0.502	9.02	40.07	40.77	2.16
FF-L (*n* = 9)	0.615	8.66	40.43	40.47	2.12
Reduced-fat cheeses					
Mean (*n* = 54)	0.460	8.98	39.62	40.88	2.14
RF-C (*n* = 9)	0.467	9.16	39.12	42.02	2.20
RF-L (*n* = 9)	0.456	8.86	38.75	42.23	2.24
RFM-C (*n* = 9)	0.478	8.84	39.26	40.81	2.16
RFM-L (*n* = 9)	0.423	8.97	39.93	40.23	2.11
RFD-C (*n* = 9)	0.461	8.92	40.14	40.09	2.09
RFD-L (*n* = 9)	0.475	9.13	40.53	39.91	2.05
SE	0.0285	0.3976	1.071	1.275	0.124
LSD	0.0655	1.181	3.182	3.788	0.367
Days of ripening					
7 (*n* = 18)	0.215 ^a^	8.73 ^a^	32.02 ^a^	45.71 ^b^	2.68 ^c^
30 (*n* = 18)	0.458 ^b^	8.59 ^a^	42.16 ^b^	39.74 ^a^	2.03 ^b^
60 (*n* = 18)	0.707 ^c^	9.61 ^b^	44.69 ^c^	37.20 ^a^	1.72 ^a^
SE	0.0162	0.281	0.757	0.902	0.087
LSD	0.0463	0.835	2.251	2.679	0.260

^1^ FF cheeses were not included in the statistical analysis. ^a–c^ Different letters within columns indicate statistically significant differences.

**Table 6:** Organoleptic evaluation of full-fat and reduced-fat sheep milk cheeses at 60 days of ripening. Cheese codes are according to Table 1 and Table 2.

Cheese	Appearance ^1^	Texture ^2^	Flavour ^3^	Total score ^4^
FF (*n* = 6)	8.3 ^a^	35.2 ^a^	40.1 ^a^	83.6 ^a^
RF (*n* = 6)	9.2 ^b^	36.7 ^b^	43.8 ^b^	89.6 ^c^
RFM (*n* = 6)	8.8 ^b^	36.1 ^a,b^	41.5 ^a^	86.4 ^b^
RFD (*n* = 6)	9.1 ^b^	36.8 ^b^	40.4 ^a^	86.3 ^a,b^
Brine				
C (*n* = 12)	8.9	36.7 ^b^	42.0	87.6 ^b^
L (*n* = 12)	8.8	35.7 ^a^	40.9	85.4 ^a^

^1^ 0–10, ^2^ 0–40, ^3^ 0–50, ^4^ 0–100 points scale. ^a–c^ Different letters within columns indicate statistically significant differences.

## Abbreviations

a_w_	water activity
β-lg	β-lactoglobulin
FDM	fat on dry matter
FF-C or FF-L	full-fat cheese salted with NaCl or NaCl/KCl, respectively
MNFS	moisture on non-fat substances
MWP	microparticulated whey protein
PDM	Protein on dry matter
RF-C or RF-L	reduced-fat cheese salted with NaCl or NaCl/KCl, respectively
RFD-C or RFD-L	reduced-fat cheese from high-pasteurized milk salted with NaCl or NaCl/KCl, respectively
RFM-C or RFM-L	reduced-fat cheese with MWP salted with NaCl or NaCl/KCl, respectively
S/M	salt in moisture
